# LETM1 Knockdown Promotes Autophagy and Apoptosis Through AMP-Activated Protein Kinase Phosphorylation-Mediated Beclin-1/Bcl-2 Complex Dissociation in Hepatocellular Carcinoma

**DOI:** 10.3389/fonc.2020.606790

**Published:** 2021-01-21

**Authors:** Baoyong Zhou, Changhong Yang, Xiong Yan, Zhengrong Shi, Heng Xiao, Xufu Wei, Ning Jiang, Zhongjun Wu

**Affiliations:** ^1^Department of Hepatobiliary Surgery, the First Affiliated Hospital of Chongqing Medical University, Chongqing, China; ^2^Department of Bioinformatics, Chongqing Medical University, Chongqing, China; ^3^Department of Pathology, Chongqing Medical University, Chongqing, China

**Keywords:** hepatocellular carcinoma, LETM1, apoptosis, autophagy, AMP-activated protein kinase (AMPK), beclin-1/Bcl-2 complex

## Abstract

Leucine zipper/EF hand-containing transmembrane-1 (LETM1) is an inner mitochondrial membrane protein that has been reported to be involved in many primary tumors and may regulate many biological processes. However, the biological role and molecular mechanism of LETM1 in the progression of hepatocellular carcinoma (HCC) remain largely unknown. In this study, we found that LETM1 was highly expressed in HCC tissues and cell lines and that higher LETM1 expression was associated with a lower overall survival rate in HCC patients. In addition, knockdown of LETM1 inhibited proliferation and enhanced apoptosis and autophagy in the Huh 7 and QGY-7701 liver cancer cell lines. Mechanistically, knockdown of LETM1 dissociated the Beclin-1/Bcl-2 complex through phosphorylation of AMPK and Bcl-2. These results demonstrated that LETM1 is involved in the development of HCC and could be a novel therapeutic target in HCC.

## Introduction

Hepatocellular carcinoma (HCC) is the sixth most common malignant tumor worldwide and is particularly prevalent in China, where approximately half of new cases and deaths occur (1, 2). Due to the high invasiveness and high mortality rate of HCC, the survival time of most patients with advanced HCC is only approximately 2–3 months (3). Given the advances in some novel therapeutic strategies against HCC such as resection, chemotherapy, and transplantation, the survival of HCC patients has improved. Unfortunately, because there are no obvious clinical symptoms at an early stage, most patients are diagnosed at an advanced stage, and the 5-year recurrence rate after surgery is greater than 70%; moreover, recurrence often results in death (4, 5). Therefore, further studies on diagnostic and prognostic markers for HCC are key to developing more effective treatments.

Autophagy is an important lysosomal process for removing damaged organelles/proteins and limiting genomic instability (6). Autophagy can regulate cellular processes such as proliferation and apoptosis in liver cells (7). Autophagy plays a variety of roles in maintaining the stability of the internal environment of the liver, maintains the genomic stability of liver cells and prevents malignant transformation (8, 9). Researchers have found that autophagy plays a key role in the adaptation of HCC cells and that dysregulation of autophagy is connected with the occurrence of liver cancer (10). Low expression of Beclin1 in human HCC tissues is associated with tumor recurrence (11). Beclin1 is an important autophagy protein that has been shown to be related to HCC tumors. Recently, a study suggested that disruption of the Beclin1/Bcl-2 complex is an effective mechanism for increasing mammalian autophagy, thus preventing premature aging (12). Both Bcl-2 and Bcl-2L1 directly bind to Beclin-1 through their BH3 domains (13). Dissociation of Bcl-2 or Bcl-2 L1 from Beclin-1 may be a necessary condition for upregulating autophagy both *in vitro* and *in vivo* (14). In addition, posttranslational modification of Beclin-1 can promote its dissociation from the Bcl-2 or Bcl-2 L1 complex (15). AMP-activated protein kinase (AMPK) has been implicated in cell growth and proliferation (16). It has been demonstrated that AMPK promotes autophagy and apoptosis, and AMPK may be upstream of Beclin1 and Bcl-2 (17). Therefore, we speculated that AMPK may regulate autophagy and apoptosis through the Beclin-1/Bcl-2 complex.

Leucine zipper/EF hand-containing transmembrane-1 (LETM1), which is a transporter protein localized to the inner mitochondrial membrane, is reported to be conserved between yeast and humans (18). Studies have shown that LETM1 has important roles in mitochondrial morphology and mitochondrial K^+^ and Ca^2+^ homeostasis (19, 20). Accumulating evidence indicates that LETM1 expression is markedly increased in various tumors and may be a potential marker for tumorigenesis (21–24). In addition, it has been demonstrated that knockdown of LETM1 can cause a significant decrease in ATP levels, after which the ADP or AMP/ATP ratio can be changed, and activated AMPK, which is a bioenergy sensor that leads to the formation of autophagosomes, is then activated (25). These findings provide a theoretical basis for the hypothesis that LETM1 may regulate AMPK-mediated autophagy. However, there are only a few studies about LETM1 in autophagy and apoptosis in HCC, and greater insight into the mechanisms linking LETM1 with HCC is needed.

Based on the findings to date, we hypothesized that LETM1 regulates autophagy and apoptosis through activation of AMPK-mediated Beclin-1/Bcl-2 complex dissociation in hepatocellular carcinoma. In this research, LETM1 was found to be highly expressed in HCC tissues, and the overall survival time of patients with high LETM1 expression was found to be significantly shorter than that of patients with low LETM1 expression. We also found that the level of LETM1 was higher in HCC cell lines than in the normal hepatocyte line LO2. Furthermore, silencing LETM1 inhibited HCC growth and promoted autophagy and apoptosis *in vitro* and *in vivo*. More importantly, we discovered for the first time that LETM1 may regulate the dissociation of the Beclin-1/Bcl-2 complex through activation of AMPK to influence autophagy and apoptosis in HCC.

## Materials and Methods

### Oncomine Data Extraction

Oncomine data were processed to assess the expression of LETM1 in tumor tissues and normal liver tissues. Then, we plotted survival curves to assess the diagnostic significance of LETM1.

### Clinical Specimen Collection

Tumor tissues and adjacent tissues from 90 HCC patients who underwent hepatic resection were obtained from the First Affiliated Hospital of Chongqing Medical University from 2012 to 2018. All patients agreed to provide informed consent, and the experimental protocols were approved by the local ethics committee. The relationships between the level of LETM1 and clinical features such as age, sex, tumor size, tumor, node, and metastasis (TNM) stage, death, or time of last follow-up were evaluated, as shown in **Table 1**. Next, we performed Pearson correlation analysis to evaluate the correlations between the LETM1 expression level and the clinical variables of HCC patients. In addition, we performed univariate and multivariate Cox regression analyses to evaluate the relative risk associated with LETM1 for the prognosis of HCC. Kaplan-Meier analysis was used to evaluate the overall survival of HCC patients with high LETM1 expression.

**Table 1:** Relationship between LETM1 expression and clinicopathologic features in patients with hepatocellular carcinoma.

Variable	LETM1 expression		p-value
	High-LETM1	Low-LETM1	
**In general**			
Adjacent tissue	30	60	0.000
Tumor tissue	53	37	
**Gender**			
Male	28	17	0.520
Female	25	20	
**Age, years**			
≤60	39	30	0.408
≤60	14	7	
**Tumor size, cm**			
≤5	18	27	0.000
≤5	35	10	
**AFP, ng/ml**			
≤400	18	19	0.099
≤400	35	18	
**HBsAg**			
Positive	24	16	0.848
Negative	29	21	
**Liver cirrhosis**			
Yes	21	12	0.486
No	32	25	
**Portal vein emboli and metastasis**			
Yes	32	12	0.009
No	21	25	
**TNM stage (AJCC)**			
I–II	19	23	0.014
III–IV	34	14	
**Tumor differentiation**			
I–II	34	25	0.737
III–IV	19	12	

### Cell Line Culture

HCC cell lines, including SMMC-7721, HCC-LM3, Huh7, HepG2, QGY-7701, and Hep3B, and the normal human liver cell line LO-2 were obtained from the Department of Hepatobiliary Surgery of the First Affiliated Hospital of Chongqing Medical University. All cells were maintained in RPMI-1640 medium (Gibco, USA) supplemented with 10% fetal bovine serum (Gibco, USA), 1% penicillin, and 1% streptomycin at 37°C in 5% CO_2_.

### Real-Time Quantitative PCR

Total RNA was extracted from tissues or cells with TRIzol reagent (Takara, Japan) according to the instructions and was then reverse transcribed into complementary DNA (cDNA), which was used as the template for PCR amplification. The 20 μl reaction system contained 1 μl of cDNA template, 0.5 μl of each primer, 10 μl of 2 × PCR mix, and 8 μl of diethylpyrocarbonate (DEPC) water. The PCR conditions were as follows: predenaturation at 95°C for 5 min, followed by 35 cycles of denaturation at 95°C for 30 s, annealing at 55°C for 30 s, and extension at 72°C for 60 s. The method was used to analyze the data.

### Western Blotting

Radioimmunoprecipitation assay buffer (RIPA) buffer purchased from Beyotime Biotechnology (China) was used to extract total protein from tissues and cultured cells, and the supernatant was collected after centrifugation (12,000×g, 10 min, 4°C). After blocking with 5% skim milk, the membrane was incubated with primary antibodies against LETM1 (1:500, Santa Cruz, USA), AMPK (1:500, ABclonal, China), p-AMPK (1:500, ABclonal, China), Bcl-2 (1:500, ABclonal, China), p-Bcl-2 (1:500, Proteintech, USA), Bax (1:500, ABclonal, China), caspase-3 (1:500, Proteintech, USA), Beclin-1 (1:500, Proteintech, USA), p62 (1:500, Proteintech, China), LC3 (1:500, Proteintech, USA), and β-actin (1:2000, ABclonal, China) overnight. The membrane was then incubated with secondary antibodies (1:2,000, Proteintech, China) for 2 h the next day. The protein bands on the blot were analyzed.

### Immunohistochemistry and Determination of Staining Results

All specimens were fixed with 4% neutral formaldehyde, embedded in paraffin, and sectioned at 4 μm. Sections were baked at 60°C for 1 h and were then dewaxed, rehydrated through a gradient alcohol series and washed. Sections were boiled in antigen repair solution for 10 min for antigen repair, and the remaining steps were carried out in accordance with the instructions. After DAB staining, the sections were counterstained with hematoxylin and sealed. The percentage of positive tumor cells was scored as follows: 1–25%, 1; 26–50%, 2; 51–75%, 3; and 76–100%, 4. The intensity of LETM1 staining was scored as follows: no staining, 0; weak staining, 1; and strong staining, 2. These two scores were multiplied to obtain the final score, and the expression of LETM1 was determined as low expression (score <4) or high expression (score ≥ 4).

### Immunofluorescence Analysis

Sterile coverslips were placed in a 24-well plate, and HCC cells were seeded on the coverslips. When the HCC cells had grown to approximately 40% confluence, they were washed with cold PBS, fixed with 4% paraformaldehyde, permeabilized with 0.3% Triton X-100 for 20 min, sealed at room temperature with 3% BSA for 1 h, and incubated with a primary antibody against LC3 (1:500, Proteintech, USA) at 4°C overnight. The next day, the plate was reheated for half an hour, washed with PBS, and incubated with secondary antibodies. Nuclei were stained with DAPI (ABclonal, China). Finally, the fluorescence staining in the cells was observed under an Olympus confocal fluorescence microscope.

### Establishment of Stable LETM1-Knockdown Cell Lines

LETM1 short hairpin RNA (shRNA) and control shRNA constructs were designed and synthesized by GenePharma Corporation (Shanghai, China). Huh7 and QGY-7701 HCC cells were transfected with LETM1-lentivirus shRNA (sh-LETM1) and control-lentivirus shRNA (sh-NC) using siLentFect™ Lipid Reagent (Bio-Rad, USA) according to the manufacturer’s protocol. Stable cell lines were established and selected with 2 μg/ml puromycin (Vicmed, China) for 8–12 days. The expression levels in the stably transfected and control HCC cells were determined by RT-qPCR and Western blotting.

### Cell Proliferation Assay

A Cell Counting Kit (CCK)-8 (Beyotime Biotechnology, China) was used to assess the effect of LETM1 on cell proliferation. Cells in different groups were seeded into 96-well plates. According to the protocol, CCK-8 solution in serum-free medium (10 μl/100 μl) was added to each well and incubated at 37°C for 2 h. The absorbance at 450 nm was measured to assess cell proliferation.

### Flow Cytometric Analysis of Apoptosis

An Annexin V-FITC/PI Apoptosis Detection Kit (Beyotime, China) was used to assess apoptosis. Cells were washed with cold PBS and resuspended in buffer. Annexin V-FITC solution was added to the cells and incubated for approximately 15 min at room temperature. Next, 5 μl of propidium iodide (PI) solution was mixed with the cells for 5 min in the dark, and the ratio of apoptotic cells was determined by flow cytometry.

### Coimmunoprecipitation

Total protein was harvested from cultured HCC cells. According to the manufacturer’s instructions, coimmunoprecipitation (Co-IP) was conducted, and an anti-Bcl-2 antibody (1:500, ABclonal, China), an anti-Beclin-1 antibody (1:500, Proteintech, USA), and protein A/G agarose (MedChemExpress, USA) were used. Immunoprecipitated proteins were collected and then used for Western blot analysis to evaluate the interaction between Beclin-1 and Bcl-2.

### Tumorigenicity Assay *In Vivo*

All animal experimental procedures were approved by the Laboratory Animal Ethics Committee of Chongqing Medical University. Cells transfected with shRNAs (2 × 10^7^ cells/ml) were injected subcutaneously into the left dorsal region of each immunodeficient mouse. The nude mice (18~22 g, 4~6 weeks old) were randomized into four groups: the sh-LETM1- Huh7 group, sh-NC- Huh7 group, sh-LETM1- QGY-7701 group, and sh-NC- QGY-7701 group (n=6 mice per group). The tumor volume (V) was calculated using the following formula: V (cm^3^) = 0.5×width^2^ (cm^2^)×length (cm). The volumes of tumors in the nude mice were recorded, and tissues were collected after 4 weeks.

### Statistical Analysis

All quantitative experimental data are shown as the means ± SEMs and were analyzed with SPSS 22 (IBM, USA) and GraphPad Prism 7.0 (CA, USA). The chi-square test and Pearson correlation analysis were used to evaluate the correlations between LETM1 expression and clinical variables of HCC patients. A Cox regression model was used for statistical analysis of survival-related factors in univariate analysis and multivariate analysis. The Kaplan-Meier method was used to generate survival curves, and the log-rank test was used to calculate differences in overall survival times. Comparisons between two groups were analyzed by t tests, and comparisons among multiple groups were analyzed by one-way ANOVA. Differences with *p* < 0.05 were considered significant.

## Results

### LETM1 Was Significantly Upregulated in Hepatocellular Carcinoma Tissues

**Figure 1:**
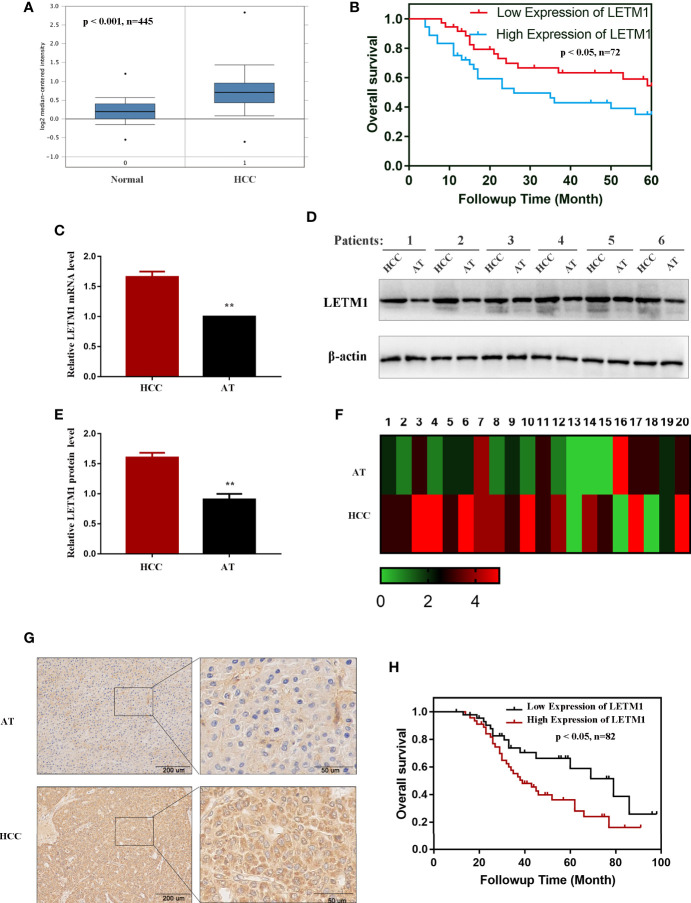
LETM1 was significantly upregulated in hepatocellular carcinoma (HCC) tissues. **(A)** Expression of LETM1 was significantly upregulated in HCC tumor tissues compared with normal liver tissue samples in the Oncomine database (*p <* 0.001, n=445). **(B)** Kaplan-Meier overall survival curves for patients with HCC stratified by high and low expression of LETM1 from the Oncomine database (*p <* 0.05, n=72). **(C)** Relative mRNA level of LETM1 in HCC tissues and adjacent tumor tissues collected from First Affiliated Hospital of Chongqing Medical University. **(D, E)** Western blot of LETM1 expression in HCC tissues and adjacent tumor tissues from First Affiliated Hospital of Chongqing Medical University. **(F)** LETM1 expression was markedly increased in 20 paired HCC tissues and their adjacent tumor tissues. **(G)** Representative images of LETM1 expression in HCC tissues and their adjacent normal tissues. **(H)** Kaplan-Meier overall survival curves for patients with HCC stratified by high and low expression of LETM1 from clinical specimens (p < 0.05, n=82). Data are presented as means ± SEM, **p < 0.01 *versus* HCC group. HCC, hepatocellular carcinoma tissue; AT, adjacent tumor tissue.

The messenger RNA (mRNA) levels of LETM1 in HCC tissues and normal liver tissues from the Oncomine database are shown in **Figure 1A**, and Kaplan-Meier survival analysis indicated that upregulated LETM1 expression predicted poor prognosis (**Figure 1B**) (*p* < 0.05). Then, the mRNA and protein levels of LETM1 in six HCC patient tissues and paired adjacent tumor tissues from the First Affiliated Hospital of Chongqing Medical University were determined by RT-qPCR and Western blotting. The results revealed that both the relative mRNA level (*p* < 0.01, **Figure 1C**) and the protein expression level (*p* < 0.01, **Figures 1D, E**) of LETM1 in HCC tissues were significantly higher than those in adjacent tumor tissues. Our results demonstrated that LETM1 overexpression may be related to HCC development.

### High LETM1 Expression Was Associated With Poor Prognosis in Hepatocellular Carcinoma Patients

Then, to illustrate the relationships between LETM1 expression and clinicopathological features as well as overall survival time in HCC patients, immunohistochemistry was conducted to detect the expression of LETM1 in 82 HCC patients (eight patients’ clinical data and follow-up data were unavailable). The data revealed that the expression of LETM1 in HCC tissues was higher than that in the corresponding adjacent tissues and that LETM1 was mainly localized in the cytoplasm, as shown in **Figures 1F, G**. Moreover, the chi-square test and Pearson correlation analysis showed that LETM1 overexpression was significantly correlated with tumor size (*p*<0.001), portal vein emboli, metastasis (both *p*<0.01), and TNM stage of HCC (*p*<0.05) (**Tables 1** and **2**). In addition, we performed univariate and multivariate Cox regression analyses to identify whether LETM1 is a risk factor in HCC patients. As shown in **Table 3**, univariate Cox regression analysis illustrated that high LETM1 expression was associated with a significantly increased risk of death in HCC patients (*p*<0.001) compared to that of patients with low LETM1 expression, and multivariate Cox regression analysis demonstrated that LETM1 expression could be a factor related to poor survival. Kaplan-Meier analysis also indicated that HCC patients with high levels of LETM1 were more likely to have a poor prognosis (p < 0.05, **Figure 1H**). Collectively, these results indicated a significant correlation of LETM1 expression with prognosis.

**Table 2:** Spearman analysis of correlation between LETM1 and clinicopathological.

Variables	LETM1 expression level	
	Spearman correlation	*p*-Value
Gender	-0.068	0.526
Age, years	0.087	0.414
Tumor size, cm	0.384	0.000
AFP, ng/ml	0.174	0.101
HBsAg	0.020	0.850
Liver cirrhosis	0.073	0.492
Portal vein emboli and metastasis	0.275	0.009
TMN stage (AJCC)	0.260	0.014
Tumor differentiation	0.035	0.741

**Table 3:** Univariate and multivariate analyses of various prognostic parameters in patients with hepatocellular carcinoma (HCC) Cox-regression analysis.

LETM1	Univariate analysis			Multivariate analysis		
	*p*	Hazard ratio	95% confidence	*p*	Hazard ratio	95% confidence
Tumor size	0.000	5.110	2.570–10.160	0.008	2.742	1.303–5.768
Portal vein emboli and metastasis	0.000	6.656	3.321–13.341	0.001	3.676	1.711–7.898
TNM stage (AJCC)	0.000	4.092	2.124–7.883	0.009	2.523	1.258–5.059

### Knockdown of LETM1 Inhibits the Proliferation and Promotes the Apoptosis of Huh7 and QGY-7701 Cells

**Figure 2:**
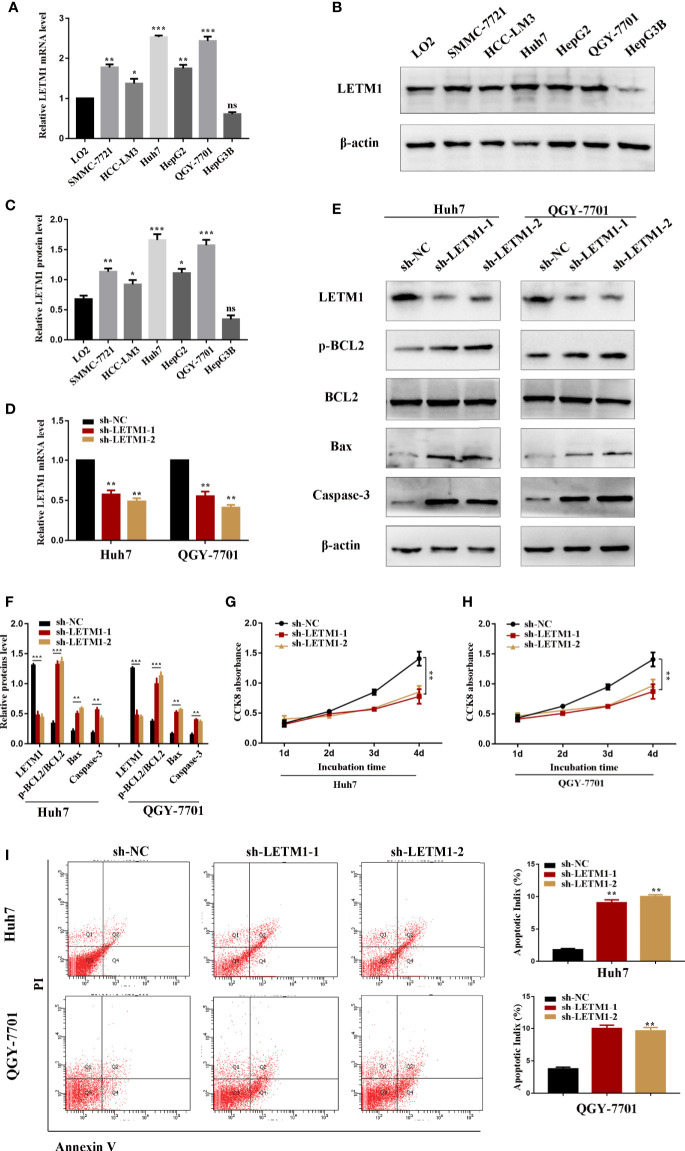
Knockdown of LETM1 inhibits cell proliferation and promotes apoptosis in hepatocellular carcinoma (HCC) cell lines. **(A)** RT-qPCR analysis of LETM1 expression in LO2 and HCC cell lines. **(B, C)** Western blotting of LETM1 expression in LO2 and HCC cell lines. **(D)** RT-qPCR analysis to estimate the transfection effect of LETM1 shRNA lentivirus. **(E, F)** Western blotting of LETM1 and apoptotic-related proteins (p-Bcl-2, Bcl-2, Bax, caspase-3) in Huh7 and QGY-7701 cell lines. **(G, H)** CCK8 assays to detect the growth rate of LEMT1 knockdown in Huh7 and QGY-7701 cell lines. **(I)** Flow cytometry to test the apoptotic index in LETM1-knockdown Huh7 and QGY-7701 cells. *p < 0.05, **p < 0.01, ***p < 0.001 *versus* sh-NC group. ns, no significance.

The level of LETM1 in LO2 normal liver cells and HCC cell lines, including SMMC-7721, HCC-LM3, Huh7, HepG2, QGY-7701, and Hep3B, was further explored by RT-qPCR and Western blotting. Compared with that in LO2 cells, the relative mRNA level of LETM1 (**Figure 2A**) was increased in SMMC-7721 (*p*<0.01), HCCLM3 (*p*<0.05), Huh7 (*p*<0.001), HepG2 (*p*<0.01), and QGY-7701 (*p*<0.001) cells, but the difference was not obvious in Hep3B cells. The Western blot results also indicated a consistent conclusion (**Figures 2B, C**). As a result, the Huh7 and QGY-7701 HCC cell lines were selected for the following experiments. Here, we used shRNA lentivirus to establish stable Huh7 and QGY-7701 LETM1-knockdown cell lines, and RT-qPCR and Western blotting were used to detect the transfection efficiency (**Figures 2D–F**). Next, the effects of LETM1 knockdown on the proliferation and apoptosis of HCC cells were explored. The CCK-8 assay revealed that proliferation was significantly decreased in the sh-LETM1 group compared with the NC group in both Huh7 and QGY-7701 cells (**Figures 2G, H**). In addition, the flow cytometry results showed that the apoptosis rate of Huh7 and QGY-7701 cells was significantly increased in the sh-LETM1 groups compared with the sh-NC groups (**Figure 2I**). The Western blot results revealed that the p-Bcl-2/Bcl-2 ratio and the expression levels of Bax and Caspase-3 were increased in the sh-LETM1 group compared with the NC group in both Huh7 and QGY-7701 cells (**Figures 2E, F**). These results indicated that knockdown of LETM1 inhibits the proliferation and promotes the apoptosis of Huh7 and QGY-7701 cells.

### Knockdown of LETM1 Triggers Autophagy in Huh7 and QGY-7701 Cells

**Figure 3:**
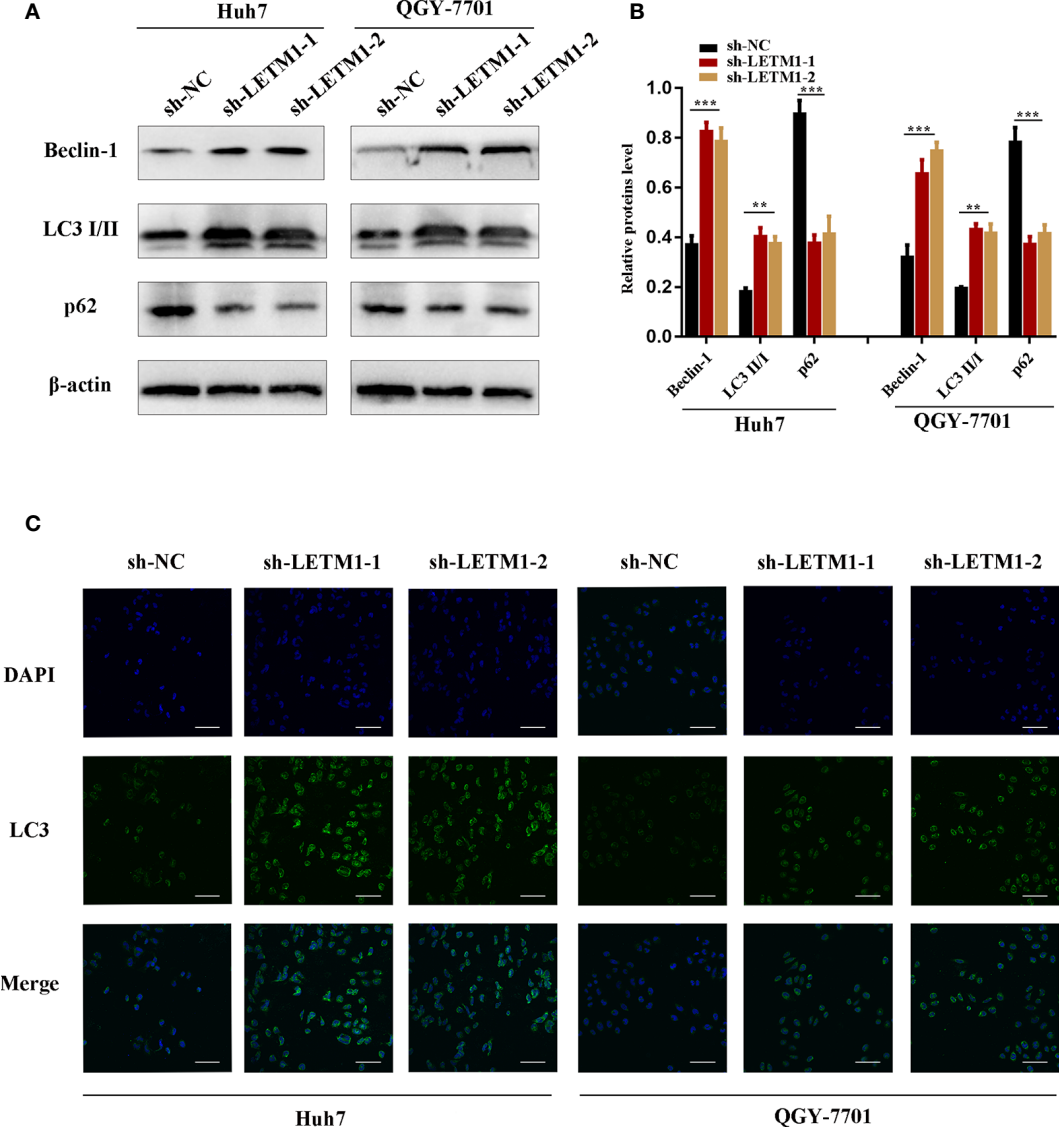
Knockdown of LETM1 triggers autophagy in Huh7 and QGY-7701 cell lines. **(A, B)** Western blotting of LETM1 and auto-related proteins in LETM1-knockdown Huh7 and QGY-7701 cell lines. **(C)** Immunofluorescence assay of LC3 to reflect autophagy level (× 400). **p < 0.01, ***p < 0.001.

Then, Western blotting and immunofluorescence were used to test whether LETM1 is involved in autophagy *in vitro*. Beclin1, p62, and LC3II/I ratio are effective markers of autophagy, and the Western blot results shown in **Figures 3A, B** revealed that the level of Beclin1 and LC3II/I were increased in the sh-LETM1 group compared with the NC group (**Figures 3A, B**), while the level of p62 was decreased in the sh-LETM1 group compared with the NC group. The immunofluorescence assay demonstrated that the fluorescence intensity of LC3 was significantly higher in the sh-LETM1 group than in the NC group in both Huh7 and QGY-7701 cells (**Figure 3C**).

### Knockdown of LETM1 Regulates Autophagy and Apoptosis by Activating AMPK

**Figure 4:**
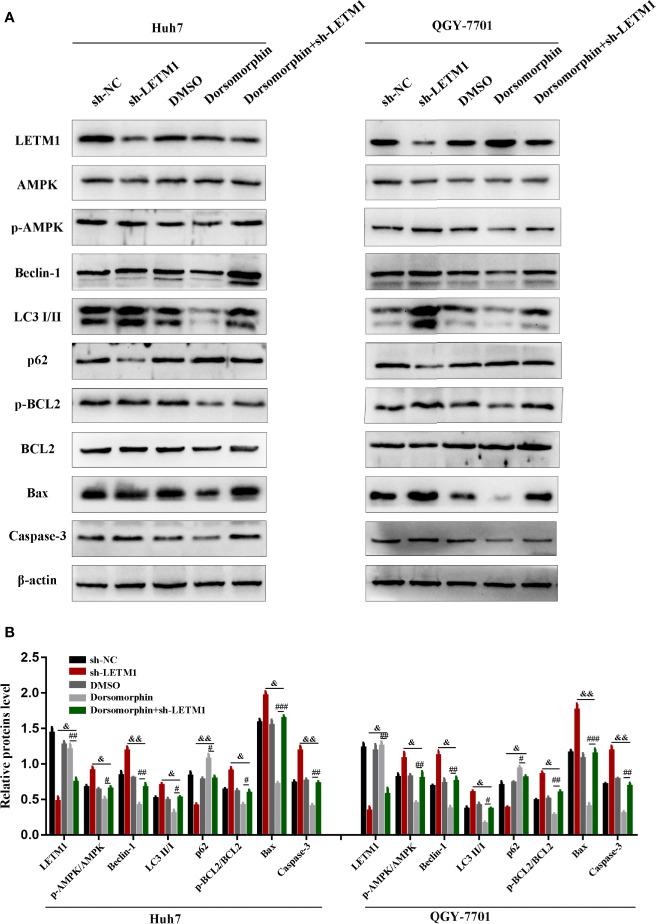
Knockdown of LETM1 regulates autophagy and apoptosis through activating AMPK. **(A, B)** Western blot analysis of the expressions of autophagic associated proteins (Beclin1, p62, and LC3), apoptotic-related proteins (p-Bcl-2, Bcl-2, Bax, caspase-3), LETM1, and AMPK in LETM1-knockdown along with AMPK inhibitor Huh7 and QGY-7701 cell lines. ^#^*p* < 0.05, ^##^*p* < 0.01, ^###^*p* < 0.001 means dorsomorphin *versus* LETM1 shRNA+dorsomorphin group; ^&^*p* < 0.05, ^&&^*p* < 0.01, means LETM1 shRNA group *versus* LETM1 shRNA+dorsomorphin group.

It has been reported that LETM1 can inhibit the activation of AMPK, and AMPK has been reported to be associated with autophagy and apoptosis. Therefore, we attempted to determine whether LETM1 can regulate autophagy and apoptosis by activating AMPK in HCC cell lines. Here, an AMPK inhibitor, dorsomorphin, together with LETM1 shRNA, was used in Huh7 and QGY-7701 cells to investigate the possible mechanism. As shown in **Figure 4**, the expression of LETM1 did not differ significantly between the sh-LETM1 group and the sh-LETM1+dorsomorphin group, while the expression of p-AMPK was increased in the sh-LETM1 group compared with the NC group, and the effect of sh-LETM1 on p-AMPK was reversed by dorsomorphin. These results indicated that LETM1 may be upstream of AMPK and that knockdown of LETM1 can promote the phosphorylation of AMPK. In addition, dorsomorphin reversed the effect of LETM1 shRNA on the expression of Beclin1, p62, and LC3II/I (LETM1 shRNA group *vs.* LETM1 shRNA+dorsomorphin group). These results demonstrated that LETM1 may regulate autophagy through AMPK phosphorylation in HCC cells.

### LETM1 Knockdown Promotes Autophagy and Apoptosis Through AMPK-Mediated Beclin-1/Bcl-2 Complex Dissociation in Hepatocellular Carcinoma Cells

**Figure 5:**
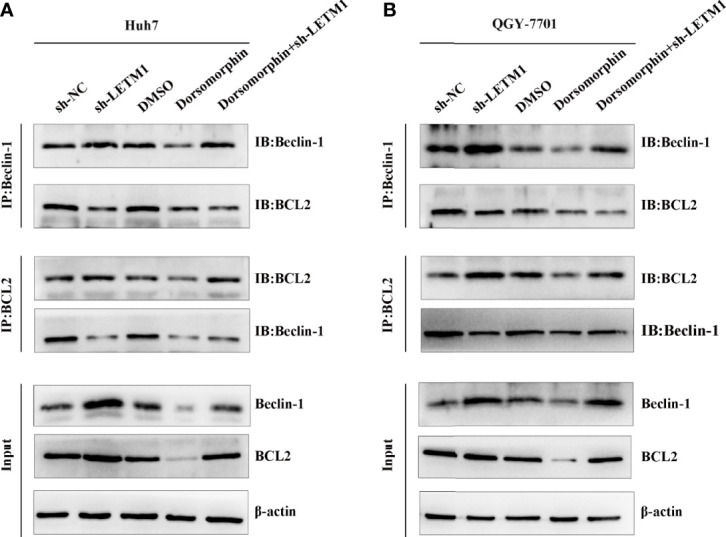
LETM1 knockdown promotes autophagy and apoptosis through AMPK mediated Beclin-1/Bcl-2 complex dissociating in hepatocellular carcinoma cell. **(A)** Co-immunoprecipitation experiment of the interation of Beclin-1 and Bcl-2 in in LETM1-knockdown along with AMPK inhibitor Huh7 cell line. **(B)** Co-immunoprecipitation experiment of the combination of Beclin-1 and Bcl-2 in in LETM1-knockdown along with AMPK inhibitor QGY-7701 cell line.

AMPK has been reported to be located upstream of Beclin-1 and Bcl-2, and Beclin-1 and Bcl-2 may exist as a complex. In addition, LETM1 can regulate the phosphorylation of AMPK. Therefore, we proposed that LETM1 regulates the dissociation of the Beclin-1/Bcl-2 complex through phosphorylation of AMPK, thus affecting autophagy and apoptosis. To verify this hypothesis, coimmunoprecipitation experiments were conducted. As shown in **Figure 5**, the interaction of Beclin-1 and Bcl-2 was decreased in the sh-LETM1 group but increased in the dorsomorphin group, indicating that LETM1 knockdown promoted the dissociation of the Beclin-1/Bcl-2 complex and that inhibition of AMPK suppressed this dissociation. Moreover, inhibition of AMPK reversed the effect of LETM1 shRNA on Beclin-1/Bcl-2 complex dissociation (LETM1 shRNA group *vs.* LETM1 shRNA+dorsomorphin group).

### LETM1 Knockdown Suppressed the Tumorigenicity of Transfected Hepatocellular Carcinoma Cell Lines *In Vivo*

**Figure 6:**
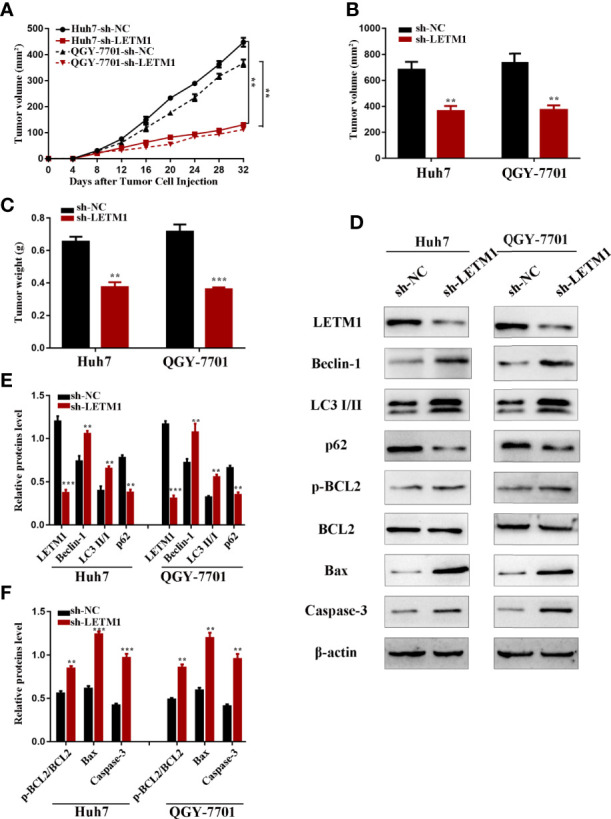
LETM1 knockdown suppressed tumorigenicity of Huh7 and QGY-7701 cells *in vivo*. **(A)** The growth curve of the *in vivo* tumorigenicity assa in LETM1 knockdown Huh7 and QGY-7701 cells transfected nude mice (N=6/per group). **(B)** The weight of tumors in LETM1 knockdown Huh7 and QGY-7701 cells transfected nude mice (N=6/per group). **(C)** The volume of tumors in LETM1 knockdown Huh7 and QGY-7701 cells transfected nude mice (N=6/per group). **(D–F)** The protein level of autophagic associated proteins (Beclin1, p62, and LC3), apoptotic-related proteins (p-Bcl-2, Bcl-2, Bax, caspase-3) and LETM1 in tumor tissues of mice. **p < 0.01, ***p < 0.001 *versus* sh-NC group.

Eventually, we further explored the effect of LETM1 knockdown on HCC growth in nude mice. Stable Huh7 and QGY-7701 LETM1-knockdown cells were injected subcutaneously into nude mice. The volume and weight of tumors were recorded; as shown in **Figure 6**, tumor growth in the sh-LETM1 group was slower than that in the NC group (*p* < 0.01) (**Figure 6A**), and the tumor weights and volumes were significantly lower in the sh-LETM1 group than in the NC group (**Figures 6B, C**). Tumor tissues were assessed by Western blotting, which revealed that the levels of Beclin1, LC3II/I, p-Bcl-2/Bcl-2, Bax, and caspase-3 were increased in sh-LETM1 tumor tissues compared with NC tumor tissues (**Figures 6D–F**). In contrast, the level of p62 was decreased in sh-LETM1 tumor tissues (**Figures 6D, E**). Taken together, these results in this tumor formation experiment indicated that LETM1 may play an important role in the tumorigenicity of HCC cells.

## Discussion

In this study, high LETM1 expression in HCC was associated with poor patient outcome. The findings of this study indicated that LETM1 is involved in HCC tumor cell aggressiveness by promoting cell proliferation and inhibiting autophagy and apoptosis. Mechanistically, this study suggested that LETM1 regulates the dissociation of the Beclin-1/Bcl-2 complex through AMPK in HCC.

The LETM1 protein has been identified as a mitochondrial membrane protein that may regulate mitochondrial morphology and cell proliferation (19, 20). There is accumulating evidence that LETM1 expression is significantly increased in several kinds of cancers and is associated with poor prognosis (22–24). Doonan et al. (20). found that downregulation of LETM1 caused the accumulation of S-phase cells and that re-expression of LETM1 reversed the accumulation of S-phase cells, suggesting that inhibiting LETM1 may suppress cell proliferation by disrupting cell cycle progression (23). Piao et al. (21). found that overexpression of LETM1 could cause the necrosis and death of HeLa cells by reducing ATP production and mitochondrial biogenesis (21). This finding was consistent with our results, which revealed that the expression of LETM1 was increased in HCC tissues and cell lines and that high expression of LETM1 was associated with tumor size, portal vein emboli, metastasis, and TNM stage in HCC patients. Therefore, these results provide evidence that LETM1 may act as a biomarker in HCC.

Autophagy has been reported to play important roles in the occurrence and development of HCC, but this possibility remains controversial (26). The conventional view is that in the early stage of liver cancer, when liver cells are stressed and DNA is damaged, autophagy can function as a tumor-suppressive mechanism by removing damaged mitochondria or liver cells with genetic mutations to maintain the genomic stability of liver cells (27, 28). After the formation of liver cancer, the system regulating autophagy is also disrupted (29). Therefore, autophagy plays a more important role in promoting the survival of liver cancer cells during tumor development than prior to tumor formation. Apoptosis resistance is considered to be another major factor affecting the occurrence of cancer (30). During hepatocarcinogenesis, the balance between cell growth and apoptosis is disrupted (31). It has been reported that LETM1 could be related to autophagy (20). In this study, we found that the levels of autophagy and apoptosis were both increased in Huh7 and QGY-7701 cells with LETM1 silencing, indicating that LETM1 knockdown promoted autophagy and apoptosis. However, further studies on the underlying mechanisms of LETM1 need to be conducted.

It has been proven that knockdown of LETM1 can lead to a significant decrease in the ATP level, resulting in changes in the ADP/ATP and AMP/ATP ratios. An abnormal AMP/ATP ratio may affect the activation of AMPK, leading to abnormal autophagosome formation (25). AMPK has been shown to be involved in mitochondrial biogenesis (32) and lysosome biogenesis (33). Studies have shown that AMPK acts as either a tumor suppressor gene or an oncogene in different cancer cells (34). Other studies have shown that AMPK-mediated signal transduction induces cancer cell death through autophagy and/or apoptosis (35). Research has confirmed that AMPK plays an important role in the activation of autophagy in HCC cells (36). In addition, it has been demonstrated that AMPK is upstream of Beclin1 and Bcl-2 and regulates autophagy and apoptosis (17), and the Beclin1/Bcl-2 complex is a key player in mammalian autophagy and apoptosis (12). Beclin-1 and Bcl-2 interact *via* their common BH3 domains (13). Conditions such as phosphorylation of Bcl-2 can cause Beclin1 and Bcl-2 to dissociate from Beclin1/Bcl-2 complex, thus regulating autophagy and apoptosis (37). The results of our study suggested that LETM1 knockdown led to dissociation of Beclin1 and Bcl-2 from the complex through phosphorylation of AMPK as well as phosphorylation of Bcl-2 and then promoted autophagy and apoptosis, respectively.

**Figure 7:**
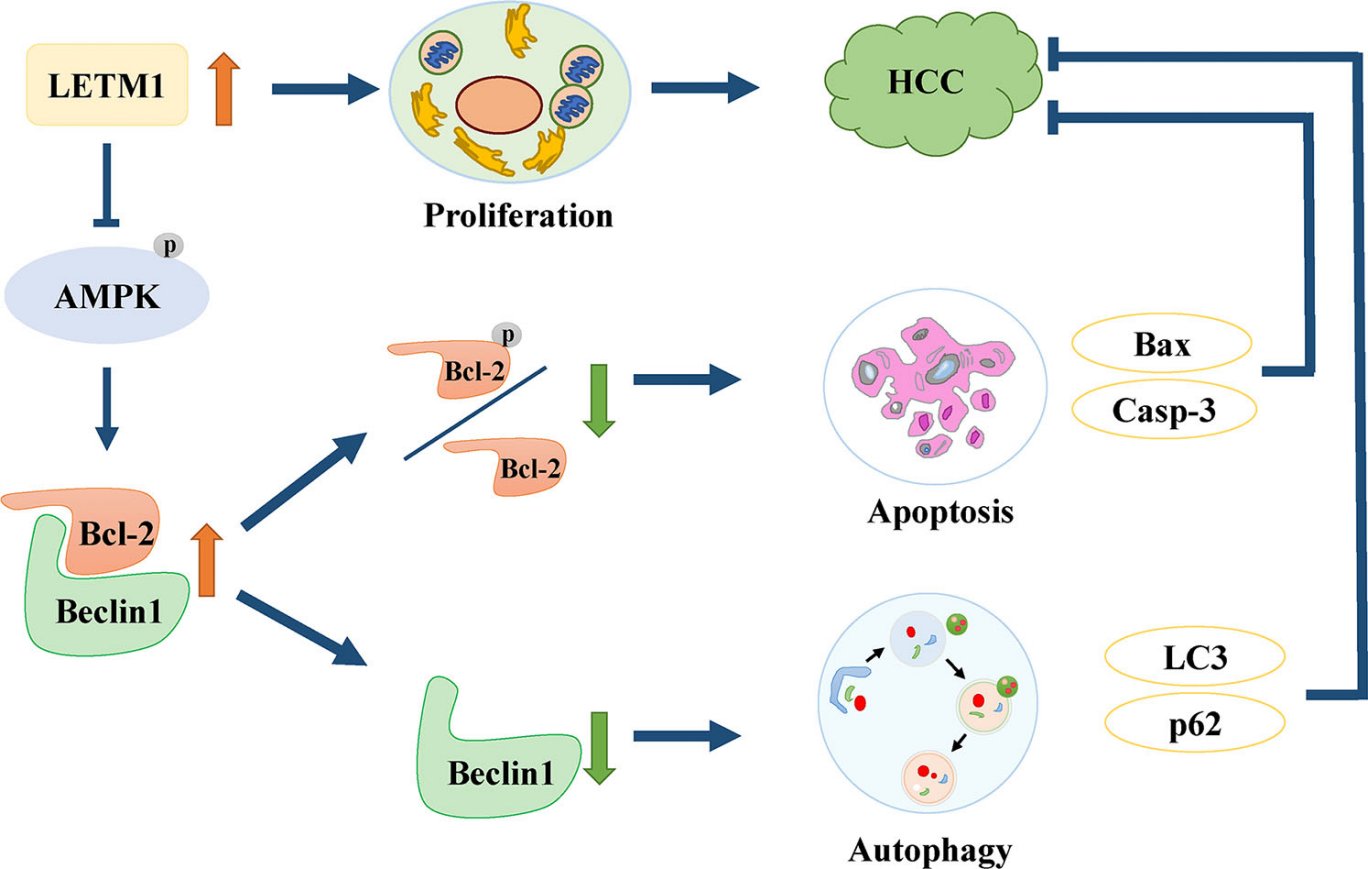
Schematic of oncogenic role of LETM1 in hepatocellular carcinoma (HCC). LETM1 promotes HCC progression through enhancing cell proliferation and suppressing autophagy and cell apoptosis *via* inhibiting AMPK activation mediated-Beclin-1/Bcl-2 complex dissociation.

In conclusion, this is the first study to find that high expression of LETM1 is significantly associated with tumor size, portal vein emboli, metastasis, TNM stage, and overall survival time in HCC patients. *In vitro* experiments demonstrated that high expression of LETM1 promoted the proliferation of HCC cells. More importantly, this is the first study to discover that high LETM1 expression inhibits autophagy and apoptosis in HCC cells. Mechanistically, LETM1 regulates autophagy and apoptosis *via* AMPK activation-mediated Beclin-1/Bcl-2 complex dissociation in HCC cells (**Figure 7**). These findings suggest that LETM1 could be a potentially valuable biomarker for the diagnosis and prognosis of HCC.

## Data Availability Statement

The raw data supporting the conclusions of this article will be made available by the authors, without undue reservation.

## Ethics Statement

The studies involving human participants were reviewed and approved by Ethics Committee of Chongqing Medical University. The patients/participants provided their written informed consent to participate in this study. The animal study was reviewed and approved by Laboratory Animal Ethics Committee of Chongqing Medical University of China.

## Author Contributions

BZ and ZW conceived and designed the experiments. CY and XY analyzed the data and processed them. HX and XW performed the experiments. NJ and ZS wrote the paper. All authors read and approved the manuscript. All authors contributed to the article and approved the submitted version.

## Funding

This work was supported by The National Natural Science Foundation of China (No. 81873592 and No. 81703063).

## Conflict of Interest

The authors declare that the research was conducted in the absence of any commercial or financial relationships that could be construed as a potential conflict of interest.

The reviewer PL declared a shared affiliation, with no collaboration, with the authors to the handling editor at the time of the review.
